# Mammographic classification of breast lesions amongst women in Enugu, South East Nigeria

**DOI:** 10.4314/ahs.v17i4.12

**Published:** 2017-12

**Authors:** Uchechukwu I Nwadike, Charles U Eze, Kelvin Agwuna, Chibuzo Mouka

**Affiliations:** 1 Department of Medical Radiography and Radiological Sciences, Faculty of Health Sciences and Technology, College of Medicine, University of Nigeria Enugu Campus, Nigeria; 2 Department of Radiation Medicine, Faculty of Medical Sciences, University of Nigeria Enugu Campus, Nigeria

**Keywords:** Mammography, breast, lesion, BI-RADS classification

## Abstract

**Objectives:**

The study was to classify lesions identified on mammograms using Breast Imaging Reporting and Data System (BIRADS) grading method. This was in view of ascertaining the rate of occurrence of breast malignancy of the studied population.

**Methods:**

A retrospective cohort study of 416 mammographic reports of women was undertaken. The reports were written by consultant radiologists of 10 years' experience and above. The reports were evaluated and characterised using Breast Imaging Reporting and Data system (BIRADS). Demographic data of patients were sourced from the request cards. The data was entered into a proforma and analysed using SPSS version 17. All request cards with incomplete data were excluded from the study.

**Results:**

Using the BI-RADS Classification, the mammographic reports shows that 29.57% of the lesions were benign, and 4.57% were suspicious and biopsy recommended, while 3.60% were highly suggestive of malignancy. The right breast was predominantly affected with 42.7% of the patients (P<0.05).

**Conclusion:**

Classification of breast lesion using BI-RADS grading system is a veritable tool in the diagnosis of the breast lesion. The present study shows that 3.6% of the population has a high index of malignancy.

## Introduction

Mammography is the use of low energy X-ray beam to examine the breast for abnormalities^1^. Mammography investigation is the most sensitive mode of breast imaging^2^. It has the ability to detect micro-calcification which cannot be delineated by clinical examination or other imaging modalities^3^.

The lesions of the breast detectable by mammograms are of two broad types; namely benign and malignant lesions. Benign lesions constitute a heterogeneous group of lesions which include developmental abnormalities, inflammatory lesions, epithelial and stromal proliferations and neoplasms^4^. These lesions' diagnosis can be accomplished with the use of mammography and thus surgery avoided because a majority of these lesions are not associated with increased risk for subsequent breast cancer^5^. Malignant lesions of the breast are divided into two based on their origin. They could be ductal or lobular breast lesions. Malignant lesions appear on mammography as micro-calcifications, irregularly shaped masses or infiltrative masses. These masses depending on their staging may fall within scale 4 – 6 of Breast Imaging Reporting Data System^6^. BI-RADS grading system of characterising breast lesions was developed by the American College of Radiology. It is a scheme for putting the finding of mammogram screening into a small number of well-defined categories. The benefit of BI-RADS to radiologists is that it forces them to critically think of the number to assign their finding thus making it possible to calculate accuracy statistically. Another advantage it offers is that the categorisation scheme has helped to standardise the words used in mammography reporting and this has reduced the confusion and improved communication between radiologists, patients, and physicians.

Mortality and morbidity due to breast cancer are on the increase around the world and Nigeria^7^. Prevalence in the US is that 1 in 8 women (12%) will develop invasive breast cancer over the course of her lifetime. In 2016, an estimated 246,660 new cases of the invasive breast cancer are expected to be diagnosed in women in the US. Mammography has been proven to detect breast cancers at an early stage and when followed up with proper clinical approach and treatment mortality from these cancers are reduced^8^. Mammography has a high sensitivity and specificity when compared with other imaging modalities such as ultrasound and magnetic resonance imaging (MRI)^4^. However, its sensitivity varies with age of the subjects^10^. For women between 50 years and above, its sensitivity is between 60 to 90% while women between 35 to 49 years have a sensitivity of 62 – 76%^10^. The sensitivity is reduced in younger women due to dense nature of their breasts^10^. The present study is aimed at characterising breast lesions of women who underwent mammography from 2010 – 2013 at University of Nigeria Teaching Hospital (UNTH), Ituku Ozalla Enugu. This is a view of classifying these lesions and identifying the current rate of breast malignancy occurrence within our locality.

## Method

A retrospective cohort study of 416 patients who were randomly selected using simple randomization technique from patients who presented for mammography from January 2010 to December 2013 was done. The study setting was the mammography section of the department of radiation medicine, University of Nigeria Teaching Hospital (UNTH), Ituku Ozalla, Enugu. The hospital is a tertiary health facility which is a centre of excellence and serves as a referral centre for special radiological examinations such as mammography. It is a catchment area for people living in the South-East geopolitical zone of Nigeria and beyond. Data reviewed were from request forms, mammographic reports, and mammographic films of patients. The data involved were the age, sex, indication for mammography, side of the breast, and mammographic findings. Request cards without relevant data such as age, clinical indication and side of breast were excluded from the study. The mammographic equipment used to obtain the images was Alpha RT manufactured by GE Hungary ZRT in the year 2009, model MGF — 101.

The mammographic examinations were done in the supero-inferior and medio-lateral projections. The radiographs were analysed by consultant radiologists of 10 years' experience and above. The lesions were classified using BI-RADS. The BI-RADS classification used in this study is in line with the American College of Radiology (ACR) recommendation^11^ as follows:

BI-RADS 0: Incomplete. Need for an additional imaging evaluation

BI-RADS 1: Normal. Normal interval follow-up

BI-RADS 2: Typically benign. Normal interval follow-up.

BI-RADS 3: Probably benign. A short interval follow-up is recommended: 4 months follow-up for masses and 6 months follow-up for micro-calcification.

BI-RADS 4: Suspicious abnormality: a biopsy should be considered.

BI-RADS 5: Highly suggestive of malignancy. Biopsy or surgery should be performed.

BI-RADS 6: Histologically proven malignancy. Imaging is performed for cancer staging or evaluation after chemotherapy.

The data was entered in a proforma and was analysed using Statistical Package for Social Science (SPSS) version 17.0 “(SPSS Incorporated Chicago, Illinois)”.

## Results

Four hundred and sixteen patients who underwent mammographic examination were reviewed.

Table 1 shows the age distribution of patients examined during the period under review. The mean age was 44.4 + 12.1 years. The highest number of patients for mammography falls within 41 – 50 years age range which is 41.1% (n=171), followed by 31 – 40 years age range 19.7% (n=82) and 51 – 60 years age range with 18.5%(n=77).

**Table 1:** Age distribution of patients

Age (Years)	Number	Percentage
11 – 20	18	4.3
21 – 30	34	8.1
31 – 40	82	19.7
41 – 50	171	41.1
51 – 60	77	18.5
61 – 70	25	6
71 – 80	9	2.1
**Total**	**416**	**100**

Source: Field survey (2013)

Table 2 shows the frequency distribution of clinical indications for which mammography was conducted. Patients with mastalgia had the highest frequency, 32.6% (n=136) followed by patients with breast lump; 24.5% (n=102). Patients who had a clinical presentation of cancer of the breast was 2.4% (n=10).

**Table 2:** Clinical indication for mammography

Clinical Indication	Number	Percentage
Breast lump	102	24.5
Breast pain	136	32.6
Nipple discharge	28	6.7
Cyst	2	0.4
Breast fullness	10	2.4
Peppery sensation	2	0.4
Mastectomy	28	6.73
Lumpectomy	12	2.8
Mastitis	4	0.9
Menopausal	2	0.4
Family history of breast cancer	6	1.4
Breast itching	8	1.92
Suspicious breast mass	2	0.4
Cancer of the breast	10	2.4
Screening	54	12.9
Total	416	100

Source: Field survey (2013)

Table 3 shows the distribution of mammographic findings and mammograms with no pathological findings, which was found to be 36.2% (n=151) and those with benign masses as 21.8% (n=91). Malignant lesions as 4.5% (n=19) and microcalcification; 3.6% (n=15) while fibroadenoma 7.6% (n=32)

**Table 3:** Mammographic findings

Mammographic finding	Number	Percentage
Normal Appearance	151	36.2
Benign tumour	91	21.8
Calcification	15	3.6
Cyst	13	3.1
Ductasia	8	1.9
Enlargement	5	1.2
Fibroadenoma	32	7.6
Lymphadenopathy	71	17.0
Malignancy	19	4.5
Veinthrombosis	2	0.4
Infections	9	2.1
Total	416	100

Source: Field Survey (2013)

Table 4 shows the right breast was significantly affected more by pathologies than the left; 42.7% (n=178) versus 34.1% (n=142) (p<0.05). Only 23.1% (n=96) of the cases affected both breasts. BI-RADS classification of the lesions identified on the mammograms was shown in table 5.

**Table 4:** Distribution of side of breast affected

Side of Breast	Number	Percentage
Right	178	42.8
Left	142	34.1
Bilateral	96	23.1
Total	416	100

Source: Field Survey (2013)

**Table 5:** BI-RADS classification of the lesions

Class	Number	Percentage
0	29	6.97
1	151	36.30
2	123	29.57
3	79	18.99
4	19	4.57
5	15	3.60
**Total**	**416**	**100**

Source: Field Survey (2013)

Class 0 to 3 show that 382 patients representing 90.83%, using the BI-RADS grading this group may be considered free from invasive breast lesions. Class 4 and 5 with a total number of 34 patients which was 8.7% represented the group with possible malignant lesions.

Table 6 represents the age bracket and the BI-RADS grading of the lesions. Grade 1 had the highest number of lesions which show normal breast mammographic appearance. Grade 3 which shows masses that are benign which may require a 4 months interval follow-up 18.99% (n=79). BI-RADS grading of 4 shows suspicious breast abnormality to be 4.57% (n=19) while grade 5 with a distribution of 3.6% (n=15) is suggestive of malignancy. The result show that age bracket of 31–70 years has a high index of malignancy but the most vulnerable age bracket is between 51—60 years.

**Table 6:** BI-RADS grading according to age.

Age (Years)	BI-RADS GRADING					
	0	1	2	3	4	5
**15 –20**	**-**	**-**	**-**	**-**	**-**	**-**
**21 – 30**	**5**	**50**	**20**	**20**	**-**	**-**
**31 – 40**	**5**	**70**	**30**	**30**	**4**	**4**
**41 – 50**	**4**	**30**	**45**	**10**	**5**	**3**
**51 – 60**	**10**	**1**	**20**	**10**	**8**	**5**
**61 – 70**	**3**	**-**	**18**	**9**	**2**	**3**
**71 – 80**	**2**	**-**	**-**	**-**	**-**	**-**
**Total**	**29**	**151**	**123**	**79**	**19**	**15**
**Percentages(%)**	**6.01**	**36.30**	**29.57**	**18.99**	**4.57**	**3.6**

Source: Field Survey (2013)

## Discussion

Mammography of four hundred and sixteen women was undertaken in this study. The study involved women between the age brackets of 15 years and above. The majority of women who were examined within the period of the study fell in within the age bracket of 41–50 years. This finding is in keeping with previous studies carried out in USA and Britain^2^,^10^. It is worthy to note that this age bracket is very vulnerable to cancer of the breast as noted in the previous studies^10^. Women with palpable masses and breast skin changes were 2.4% while it was 5% in a similar study by Wallak et al, while the study by Naku Ghartey et al had a value of 0.76%^19^,^21^. The difference in the statistics may be due to the population used in the study. The mean age for this present study was 44n.4 + 12.1 years and varied with the findings of other studies done in USA and Britain where the mean age are 61 and 50 years respectively^2^,^10^.

The mammographic investigation is undertaken following a radiological request from clinicians. In the present study, the most frequent clinical indications were breast pain (mastalgia) and breast lump or masses. Persistent breast pain will induce a conscious woman to go for the medical check; however, it is known that mastalgia which may present with some degree of inflammation rarely occurs with breast tumour^9^. Breast masses could be benign or malignant, its anatomy is usually unclear until it is subjected to mammographic investigation or biopsy^4^. Mammographic classification of the present study is 4.5% of the studied population, this figure is low when compared with the study by Pardal et al^20^.

Using BI-RADS classification, BI-RADS 0 in the present study is 6.97% (n=29). This finding is not statistically different from the finding of the study done by Bello^15^. BI-RADS 1 from this study is 36.3% (n=151), this figure differs from that of a previous study^15^. From the present study, the category that fell within BI-RADS 4 and 5 is 8.17% representing the category that is vulnerable to malignancy. This figure is much lower than the findings of a previous study which opines that 44.5% of women in Enugu are vulnerable to cancer of the breast^4^.

The present finding could well be attributed to improved breast health care adopted by most women due to increased awareness programme by various NGOs and concerned organisations.

The study revealed that right breast was mostly affected than the left with 42.8% (n=178). This result, however, differs from the finding of a previous study which opines that the left breast is most vulnerable^13^.

Micro-calcification which is a hallmark in mammographic findings in this study was 3.6% .(n=15) This finding is in keeping with the finding of a previous study^15^ of 3.28%. Malignant lesions delineated by mammography in the present study cut across the age brackets of 31 to 60 years. However, age bracket mostly affected were those within the age bracket of 51 – 60 years. This finding is in keeping with the studies done with the Caucasians^2^,^10^. This shows that age is the most recognised risk factor in breast cancer and its incidence. Abnormal mammograms from the present study are 63.8%. This finding compares favourably with finding from previous studies which range from 29% – 83%^1^,^16^,^17^.

BI-RADS classification of mammographic reports in the present study has shown that 19 subjects representing 4.5% of the population were classed in grade 4 while 15 representing 3.6% were classed in grade 5. These two grades show that 8.2% of the screened population have a high index of invasive breast lesion; this figure is low compared with the current 12% value for women in the United States^18^ but significantly lower from the studies of Adebamowo^4^, who opined that 44.5% of women in Enugu may have breast cancer. The study also revealed that age group 51–60 years is the most vulnerable amongst the age brackets. This group has higher numbers of patients with both grades 4 and 5 of the BI-RADS classification.
